# Supplementary material for: PCR for detection of Leishmania donovani from microscopically negative tissue smears of suspected patients in Gondar, Ethiopia
Source: PLoS Negl Trop Dis. 2023 Feb 13;17(2):e0011128. doi: 10.1371/journal.pntd.0011128 (PMC9956792; doi:10.1371/journal.pntd.0011128)
Supplement: S1 Table — * A bone marrow and splenic aspirate sample, both from a primary VL patient were not included in the table, as they were not positive by the HBB PCR, indicating insufficient sample or an inefficient extraction, hence they were excluded from the study. (DOCX) [file pntd.0011128.s001.docx]

**S1 Table: Overview of microscopically negative bone marrow and splenic aspirate samples from primary VL, test-of-cure (TOC), and relapse patients. *** A bone marrow and splenic aspirate sample, both from a primary VL patient were not included in the table, as they were not positive by the HBB PCR, indicating insufficient sample or an inefficient extraction, hence they were excluded from the study.

|  | **Primary VL** | **TOC** | **Relapse** | **Total** |
| --- | --- | --- | --- | --- |
| **Spleen** | 87* | 19 | 29 | 135 |
| **Bone marrow** | 37* | 10 | 9 | 56 |
| **Total** | 124* | 29 | 38 | 191 |
