# Supplementary material for: PCR for detection of Leishmania donovani from microscopically negative tissue smears of suspected patients in Gondar, Ethiopia
Source: PLoS Negl Trop Dis. 2023 Feb 13;17(2):e0011128. doi: 10.1371/journal.pntd.0011128 (PMC9956792; doi:10.1371/journal.pntd.0011128)
Supplement: S2 Table — (DOCX) [file pntd.0011128.s002.docx]

**Supplementary Table S2: Number and percentage of PCR positive and negative results among microscopically negative tissue slides, according to sample stage and type.**

| **Sampling  stage/type** | | **N micro -** | **%PCR +  micro -** | **95% CI** |
| --- | --- | --- | --- | --- |
| **Overall** *(n = 191)* | |  |  |  |
|  | PCR negative | 72 |  |  |
|  | PCR positive | 119 | 62.3% | 55.0%- 69.1% |
| **According to sample stage** | | |  |  |
| *Primary VL (n = 124)* | |  |  |  |
|  | PCR negative ^a^ | 49 |  |  |
|  | PCR positive ^b^ | 75 | 60.5% | 51.3%- ­69.0% |
| *Test of cure* *(n = 29)* | |  |  |  |
|  | PCR negative | 8 |  |  |
|  | PCR positive | 21 | 72.4% | 52.5%-86.6% |
| *Relapse* *(n = 38)* | |  |  |  |
|  | PCR negative | 15 |  |  |
|  | PCR positive | 23 | 60.5% | 43.5%- ­75.5% |
| **According to sample type** | | |  |  |
| *Bone marrow (n = 54)* | |  |  |  |
|  | PCR negative | 10 |  |  |
|  | PCR positive | 46 | 82.1% | 69.2%-90.7% |
| *Spleen (n = 135)* | |  |  |  |
|  | PCR negative | 62 |  |  |
|  | PCR positive | 73 | 54.1% | 45.3%-62.6% |

^a^ 29 (59.2%) rK39 positive, 18 (36.7%) rK39 negative, 2 (4.1%) rK39 not done
^b^ 54 (72.0%) rK39 positive, 18 (24.0%) rK39 negative, 3 (4.0%) rK39 not done
