# Supplementary material for: PCR for detection of Leishmania donovani from microscopically negative tissue smears of suspected patients in Gondar, Ethiopia
Source: PLoS Negl Trop Dis. 2023 Feb 13;17(2):e0011128. doi: 10.1371/journal.pntd.0011128 (PMC9956792; doi:10.1371/journal.pntd.0011128)
Supplement: S3 Table — POC: point of care, WBC: white blood cell, Hb: hemoglobin, Plt: platelets, N.A.: data not available or not found on the patient chart. (DOCX) [file pntd.0011128.s003.docx]

**S3 Table: Overview of PCR negative patients that were treated with antileishmanial drugs.** POC: point of care, WBC: white blood cell, Hb: hemoglobin, Plt: platelets, N.A.: data not available or not found on the patient chart.

| **Patient nr** | **Stage** | **rK39** | **Sample** | **Micro** | **Fever** | **HIV** | **Travel history** | **Splenomegaly POC** | **WBC/µL** | **Hb/dL** | **Plt/µL** | **Outcome** | **Splenomegaly discharge** |
| --- | --- | --- | --- | --- | --- | --- | --- | --- | --- | --- | --- | --- | --- |
| 1 | Primary VL | + | SA | - | Yes | N.A. | Yes | Yes - 17 cm | 1.8 | 4.8 | 74 | Cured | Yes - 8 cm |
| 2 | Primary VL | + | SA | - | Yes | Negative | Yes | Yes - 6 cm | 2.3 | 11.2 | 96 | Cured | Yes - 3 cm |
| 3 | Primary VL | + | SA | - | Yes | N.A. | Yes | Yes - 17 cm | 1.9 | 4.0 | 55 | Cured | Yes - 4 cm |
| 4 | Primary VL | + | SA | - | Yes | N.A. | Yes | Yes - N.A. | 2.5 | 6.8 | 56 | N.A. | N.A. |
| 5 | Primary VL | + | BM | - | Yes | Negative | Yes | Yes - 2 cm | 4.2 | 2.1 | 42 | Cured | No |
| 6 | Primary VL | - | BM | - | Yes | N.A. | Yes | Yes - N.A. | 2.1 | 11.4 | 38 | N.A. | N.A. |
| 7 | Relapse | N.A. | SA | - | Yes | N.A. | Yes | Yes - N.A. | 3.2 | 11.8 | 97 | N.A. | N.A. |
| 8 | Relapse | N.A. | SA | - | Yes | Negative | Yes | Yes - 10 cm | 2.5 | 13.5 | 62 | Cured | Yes - 5 cm |
| 9 | Relapse | N.A. | SA | - | Yes | Negative | Yes | Yes - 12 cm | 3.4 | 4.3 | 73 | Cured | Yes - 6 cm |
