# Supplementary material for: PCR for detection of Leishmania donovani from microscopically negative tissue smears of suspected patients in Gondar, Ethiopia
Source: PLoS Negl Trop Dis. 2023 Feb 13;17(2):e0011128. doi: 10.1371/journal.pntd.0011128 (PMC9956792; doi:10.1371/journal.pntd.0011128)
Supplement: Attachment — Submitted filename: Rebuttal letter_FINAL.docx [file pntd.0011128.s004.docx]

**Rebuttal letter**

Key Review Criteria Required for Acceptance

As you describe the new analyses required for acceptance, please consider the following:

**Methods**

Reviewer #1:

- Are the objectives of the study clearly articulated with a clear testable hypothesis stated? Yes
- Is the study design appropriate to address the stated objectives? Yes
- Is the population clearly described and appropriate for the hypothesis being tested? Yes
- Is the sample size sufficient to ensure adequate power to address the hypothesis being tested? Yes
- Were correct statistical analysis used to support conclusions? Yes
- Are there concerns about ethical or regulatory requirements being met? Yes

Reviewer #2:

(No Response)

Reviewer #3:

- Line 104-105: “random selection …varying parasite gradings…” Please mention what the parasite grades were and how these were randomly picked.

The positive slides were collected through random sampling of positive slides at convenience. The gradings were two times +1, six times +2, three times +3 and one of +4, +5 and +6. This information was added to the methods section.

- Was the sample size of 14 from microscopy positive adequate to demonstrate the difference in Ct values?

The mean goals to select the 14 microscopy positive samples was to show that the extraction and PCR from tissue smears on slides is working well. Additionally, we tested whether the Ct values of the PCR positive, microscopy negative slides were lower than the Ct values of the microscopy positive slides. Given that we were able to achieve statistical significance with 14 samples, the post-hoc power to find a true difference was likely rather high, in line with the biological explanation of this effect.

- Line 228: define severity of splenomegaly

The sentence in the manuscript was revised to “patients with a larger spleen size” to clarify the severity of splenomegaly. Moreover, between parenthesis in that sentence it is also specified as OR 1.06 for each difference of one cm, which also indicates the size of the spleen.

--------------------

**Results**

Reviewer #1:

- Does the analysis presented match the analysis plan? Yes
- Are the results clearly and completely presented? Yes
- Are the figures (Tables, Images) of sufficient quality for clarity? Yes

Reviewer #2:

(No Response)

Reviewer #3:

- It is mentioned that in 2020, 619 patients had tissue aspiration at the study site and 432 were negative. It will be clearer if a flow diagram of the study population, inclusion-exclusion is included in the paper.

We agree with the reviewer, but unfortunately the detailed information of which slides were included/excluded and the reason why were not collected during the study. This information would indeed have been useful for example to see which samples were excluded, which could have an impact on the PCR positivity proportions. Therefore, we described this limitation of the study in the discussion.

- Make sure that the + and – signs be clearly visible in the figures 1 and 2

Adapted.

- Line 220: the sample size for microscopy positive and microscopy negative are exchanged

Thank you for noticing. Adapted.

- Line 231: “At a cut-off of 50% (log-odds = 0), the sensitivity in this sample population 231 was 81%...” this statement is not clear. Please explain which variable the cut-off of 50% refers to. It is important to describe this statistical analysis in the methods section too.

We clarified in the manuscript that this refers to the cut-off of 50% probability to be PCR positive according to the model. The prediction rule gives a probability to be positive for each sample. Such rule can be evaluated in many ways. One simple way is to dichotomize it, which we do here: we evaluate the sensitivity and specificity against PCR if in clinical practice, we would consider samples with a predicted probability of over 50% to be positive, and negative otherwise.

--------------------

**Conclusions**

Reviewer #1:

- Are the conclusions supported by the data presented? Yes
- Are the limitations of analysis clearly described? Yes
- Do the authors discuss how these data can be helpful to advance our understanding of the topic under study? Yes
- Is public health relevance addressed? Yes

Reviewer #2:

(No Response)

Reviewer #3:

- PCR positivity of ToC patients (72.4%) is higher than all the other microscopy negative groups (primary and relapse). It is important to discuss this finding. Is it possible that PCR is detecting non-viable DNA?

We completely agree with the reviewer’s comments. We added a section in the discussion in which we explain that TOC PCR results should be interpreted with care as there could still be detection of kDNA while the parasite are already death or cleared. The SL-RNA qPCR (Eberhardt et al 2018) could contribute to that, although its sensitivity is slightly lower than for kDNA.

- Higher detection of VL from bone marrow smears than spleen was found in this study. Several reasons can be entertained for this finding and may be discussed. For example, it is the sicker patient who undergo bone marrow aspiration than spleen aspiration (those with bleeding, very low platelet count, ascitis). Such patients tend to have higher parasite load. This may not be reflected on parasite grading which is typically based on spleen aspiration. Bone marrow samples tend to be more in volume and diluted than spleen aspirates. Higher chance to miss on microscopy due to diluted bone marrow sample; and scanty tissue from spleen aspirate and probably inadequate sample for PCR from the spleen slides.

For both sample types, if the kDNA PCR was negative, a HBB PCR was done to check if there was sufficient tissue on the slide and if the DNA isolation was performed successfully. Samples that did not have a HBB Ct value lower than 35 were excluded from the analysis. We agree with the reviewer however that it is the sicker patients that undergo bone marrow aspiration and that this sample is higher in volume than the splenic aspirate which can lead to dilution of the sample and make it more difficult to find the parasites under the microscope, even though they are there. This information was added in the discussion of the revised manuscript.

--------------------

**Editorial and Data Presentation Modifications**

Use this section for editorial suggestions as well as relatively minor modifications of existing data that would enhance clarity. If the only modifications needed are minor and/or editorial, you may wish to recommend “Minor Revision” or “Accept”.

Reviewer #1:

- It would be better if author clearly define the number of spleen and bone marrow samples for each individual cohort (POC, relapse and TOC) as supplemental figure.

An additional table was included in the supporting information (Table S1) to clarify how many splenic and bone marrow aspirates were included for each cohort.

Table 1: author needs to clearly define the distribution of number of cases (PCR negative) in this table in accordance with figure-1.

We are not sure whether we clearly understand the reviewer’s comment, but we think he/she is advising us to include the rK39 results also in Table 1. As there are also test-of-cure patients among the samples presented in table 1, which were not tested by rK39, this would mean 3 additional tables would have to be included in the manuscript. These are presented here below. From these tables, no association can be observed of the rK39 RDT on the predicted or true positives.

| **rK39 negative** | Predicted negative | Predicted positive |
| --- | --- | --- |
| PCR negative | 9 | 9 |
| PCR positive | 4 | 14 |

| **rK39 positive** | Predicted negative | Predicted positive |
| --- | --- | --- |
| PCR negative | 11 | 18 |
| PCR positive | 10 | 44 |

| **rK39 not done** | Predicted negative | Predicted positive |
| --- | --- | --- |
| PCR negative | 8 | 17 |
| PCR positive | 9 | 38 |

Reviewer #2:

(No Response)

Reviewer #3:

- Line 95: “small on non-palpable” – should read “small or non-palpable”

Adapted.

--------------------

**Summary and General Comments**

Use this section to provide overall comments, discuss strengths/weaknesses of the study, novelty, significance, general execution and scholarship. You may also include additional comments for the author, including concerns about dual publication, research ethics, or publication ethics. If requesting major revision, please articulate the new experiments that are needed.

Reviewer #1:

This is a very good study that could make significant impact on diagnosis and care in a resource limited settings of Ethiopia. In this study author represents three critical aspects:

- Lack of sensitivity of current diagnostic (rk39 rapid diagnostic test and microscopy of tissue smears) procedure in Gondar, Ethiopia.
- Potential of real time PCR for detection of L. donovani from microscopically negative spleen and bone marrow smears of suspected patients in Gondar, Ethiopia.
- Implementation of PCR could improve patient diagnosis and treatment at least for microscopically negative bone marrow cases.

But, there are some minor issues that deserve discussion:

- Figure 2: author represents the percentage and number of microscopically negative PCR positive bone marrow samples, not correlates with the statements in the line number 28 and 196.

Thank you for noticing this typo. We corrected it in the revised manuscript.

- Figure 3: Number of microscopically negative and positive samples (n) in the figure, not correlate with the figure legends- and with line number-104.

The numbers in the figure legend were indeed switched. Corrected.

- Line 211-212: author needs to check the number of not tested patients, not correlates with the figure-1 legend.

Thank you for noticing, the numbers were indeed reverted in the main text. Corrected in the revised manuscript.

- Author need to clearly define how they measure sensitivity and specificity.

Sensitivity and specificity were defined as the proportion of correctly predicted cases among respectively the positive and negative PCR. This was added to the statistics section in the methods.

- It would be better if author had measured the parasite load and show correlation between Ct values of PCR positive microscopically negative samples with parasite load at least for bone marrow samples.

Grading or determination of the parasite load cannot be done on microscopically negative slides, as no parasites are observed under the microscope. Therefore, making a correlation with the grading of microscopically negative slides and their respective Ct values is not possible. We could do this for the microscopically positive slides, however, since there are only 14 samples this would not make much sense.

- *Leishmania* detection: Author need to define the conc. of DNA template used for PCR study along with the volume used.

The concentration of DNA was not measured before adding the template to the mastermix. However, we have conducted an HBB PCR for PCR negative samples, as described in the manuscript, to confirm that there was enough tissue on the slides and that the extraction was successful.

According to me, this manuscript could be accepted after such changes.

Reviewer #2:

*Major Points*

- What was the final elute volume

DNA was eluted in 50 µL nuclease free water. Added to the manuscript.

- How did you decide if there was 'insufficient material' when extracting the samples (line 125)

Insufficient material was defined as less than a fifth of the slide covered by tissue. This was added to the methods section. Also, we added an additional paragraph in the discussion to describe that this is a main limitation of the study. Since splenic aspirates often have less volume, these slides were presumably (no data recorded) more frequently not included in the current study. Out study does unfortunately not allow to demonstrate whether these slides would have been positive by PCR or not, which could have affected the % positive slides. We thank the reviewer for this important insight.

- Please clarify "Whenever a sample had a high Ct-value (Ct ≥ 35), the sample was re-tested to confirm the positive result. If the sample was negative during retesting, the final result was negative." How was negative defined here? For example if I ran a sample in duplicate n the first run and got CT 36/36 - this was then re-run in duplicate again? If it amplified at 37/37 again was this positive or negative? Presumably you had a CT threshold for positive/negative.

We agree with the reviewer that this was poorly described and tried to elaborate on the methods uses in the revised manuscript. A sample was only tested once in each PCR run. When a Ct-value was under 35, the sample was considered positive. Whenever a sample had a Ct above 35, it was retested in the next run to confirm the positive run. If it was again positive in the repeat run, with any Ct value, the sample was considered positive. If the repeat was negative (no fluorescence), it was considered negative. This to exclude any false-positive results.

- The statistical analysis is not adequately described in particular outlining which variables were used in the model and how categorical varibales were constructed. Equally there are comparisons provided in the results (yield of PCR between bone marrow and splenic samples for example) which were not described in the methods. The results of the model are also not well presented. There is no sense of what variables were included with only a final model given.
  We added the variables included in the model, including the scales and/or categories in the methods section.
- I found the results quite difficult to follow mixed between the flow charts and text. Please consider revisiting this as its important information. I would strongly encourage a table stratified by status/sample type etc.

An additional supplementary table was added (Table S2) to support the flow chart in Fig 1 and to clarify the text. In a previous version sent out to all authors, we had only the tables, and then it was suggested to replace them by figures. Therefore, we prefer to keep the figures in the main manuscript and add the table in supporting information.

*Minor Points:*

- Line 70-72 - "The sensitivity of microscopic examination of tissue smears is also suboptimal ......" this sentence should be referenced.

Reference was added.

- Line 127 - 'after a quick spin'. Please clarify if you literally just mean vortexing or if you mean X minutes at Y RPM so that the method could in theory be replicated.

It was indeed a brief vortex (3 sec). Clarified in the manuscript.

Reviewer #3:

General comments:

- The study demonstrated that a high proportion of patients were missed when RDT and tissue aspirate microscopy are used. The use of PCR increased case detection. It was reported before that the diagnostic performance of rK39 RDT in east Africa is low. This finding suggests the urgent and strong need for better diagnostics for VL in the region. This needs to be emphasized in the paper.

We agree with the reviewer and have revised the discussion section, more emphasizing on the overall need for better diagnostics for VL (preferably on a non-invasive sample) in East-Africa.

- Authors showed that several rK39 RDT positive cases but tissue aspirate microscopy were positive on PCR testing. It is important to discuss if this might show that rK39 RDT actually have better performance than reported; and that lower rK39 RDT performance might be due to reference test bias (low sensitivity of tissue aspirate microscopy).

We agree with the reviewer and added a statement in the discussion about the fact that rK39 sensitivity is perhaps better than described before. However, there is also a lot of samples that are positive by rK39, yet PCR negative, which is probably the predominant reason why clinicians in northern Ethiopia do not follow the current diagnostic algorithm properly.

- It this retrospective study at a busy leishmaniasis treatment and research center, there can be easy contamination of samples during processing or storage of slides. It is important to describe what measures are place to avoid that. Is keeping empty slides between tissue smear slides a routine activity? Contamination of samples (slides) can have significant impact on the findings of such study (molecular diagnostics) and needs to be described clearly or discussed as a potential limitation.

We agree with the reviewer and added to the methods that the laboratory is GCLP compliant and twice per year internal and external quality controls are done for Leishmania detection by PCR. We have also added this to the discussion as a limitation of the study by saying “even though control slides were stored between the other slides, we can never completely rule out that there was no contamination”.

*Abstract*

- Line 25: The number and proportion of bone marrow and spleen aspirate may be included with this sentence mentioning the total sample size.

Added in the revised abstract as recommended by the reviewer.

- More strong recommendation on the need for better diagnostics for VL in the region can be provided here given the significant gaps demonstrated.

We added a sentence to the abstract to emphasize on the importance for better diagnostics for VL in East Africa.

*Author summary*

- Line 50: recommends implementing PCR at least on microscopically negative bone marrow aspirate smears. This sentence undermines the fact that fewer proportion of patient undergo bone marrow aspiration, and still the PCR positivity on spleen aspiration microscopy negative high.

We agree with the reviewer and adapted the author summary, indicating that molecular tools in general are needed to improve diagnostics for VL.

*Introduction*

- Line 59-60: States the importance of reliable diagnostic methods for treatment decision making. The prior sentences describe the magnitude of VL and its fatality. It is important, additionally, to mention the cost implications and adverse effects related to treatment. The need for reliable diagnostics is due to the fact that the complication of the disease needs to be balanced with the complication of the treatment.

We agree with the reviewer and added an argument in the introduction, describing that good diagnostics are not only needed to avoid death but also to avoid unnecessary costs and adverse effects in case of overtreatment.
