# Supplementary material for: PCR for detection of Leishmania donovani from microscopically negative tissue smears of suspected patients in Gondar, Ethiopia
Source: PLoS Negl Trop Dis. 2023 Feb 13;17(2):e0011128. doi: 10.1371/journal.pntd.0011128 (PMC9956792; doi:10.1371/journal.pntd.0011128)
Supplement: Attachment — Submitted filename: Rebuttal letter 2.docx [file pntd.0011128.s005.docx]

**Rebuttal letter 2**

In response to reviewer 3 you have said

"The positive slides were collected through random sampling of positive slides at convenience. "

Random sampling and convenience sampling are fundamentally different. Was random sampling or convenience sampling used.

The response in the rebuttal letter was incorrect. It was indeed random sampling, as written in the manuscript.

- Line 228: define severity of splenomegaly The sentence in the manuscript was revised to “patients with a larger spleen size” to clarify the severity of splenomegaly.

This doesnt really provide clarification. I think you need to either give a definition of larger or state more clearly tht you fitted spleen size as a linear variable - if so did you assess for evidence of non-lineatiy of the associations?

We agree with the reviewer that severity indeed reflects the disease, but what was measured was de enlargement of the spleen (in cm), compared to a normal spleen. However, we did not use the wording “severity of splenomegaly” in the manuscript. We revised this in the manuscript section on statistics as: “splenomegaly measured as the enlargement of the spleen compared to a normal spleen”.
